# “I want to see them thrive!”: exploring health service research priorities for young Aboriginal children growing up in Alice Springs – a qualitative study

**DOI:** 10.1186/s12913-024-10642-8

**Published:** 2024-02-15

**Authors:** C. Lloyd-Johnsen, A. Hampton, E. Stubbs, S. Moore, S. Eades, A. D’Aprano, S. Goldfeld

**Affiliations:** 1 Centre for Community Child Health, Murdoch Children’s Research Institute, Flemington Rd, Parkville, Melbourne, VIC 3052 Australia; 2 Department of Paediatrics, University of Melbourne, Melbourne, VIC Australia; 3 Central Australian Aboriginal Congress, Alice Springs, NT Australia; 4 Menzies School of Health Research, Darwin, NT Australia; 5 Flinders University, Adelaide, SA Australia; 6 Melbourne School of Population and Global Health, The University of Melbourne, Melbourne, Australia; 7 Royal Children’s Hospital, Melbourne, VIC Australia

**Keywords:** Australian Aboriginal, Indigenous, Child health, Longitudinal studies, Qualitative study, Health priorities, Community engagement, Thematic analysis

## Abstract

To better understand the specific influences of early life on the long-term health and well-being of local Aboriginal children in Alice Springs, high-quality local longitudinal data is required. The Central Australian Aboriginal Congress and the Murdoch Children's Research Institute are exploring the feasibility of establishing a cohort study to fill this gap. A nested qualitative study was conducted to identify priority issues that can be translated into research questions answerable through the proposed cohort study. Semi-structured interviews and focus group discussions (FGDs) were conducted with a range of key community stakeholders, parents and caregivers of young Aboriginal children from Alice Springs in the Northern Territory between 2020 and 2021. Two Aboriginal and two non-Aboriginal researchers conducted 27 interviews and 3 FGDs with 42 participants. Three broad themes were constructed through reflexive thematic analysis representing the areas of focus community stakeholders and parents want future research to prioritise: (1) social determinants of health (2) building positive connections, and (3) making sure kids grow up strong and healthy. Priority setting for future research should be driven by Aboriginal and Torres Strait Islander peoples in order to be of practical benefit to their community. This qualitative study found that housing, transport and positive connections through nurturing and engaged parents were some of the most important issues raised. Participants also wanted future research to focus on issues specific to children such as nutrition, hearing loss, language development and capacity to learn. These findings will guide future work led by local Aboriginal researchers to co-design the proposed cohort study.

**Supplementary Information:**

The online version contains supplementary material available at 10.1186/s12913-024-10642-8.

## Background

Australia’s First Peoples continue to experience poorer health outcomes and greater social disadvantage relative to non-Indigenous Australians [1, 2]. These well-documented inequities are exacerbated by the ongoing impact of colonisation, dispossession and disempowerment [3, 4]. Charting trajectories across critical or sensitive periods of growth and development in early life may help provide a multidimensional perspective as to why these inequities persist. To do so, we need access to high-quality repeated measurements of data on individuals collected over time [5, 6]. Having access to accurate longitudinal data on the health and well-being of Aboriginal and Torres Strait Islander children as they grow allows researchers to build a more reliable account of key events and experiences. It can help inform decision-making about service delivery and public health initiatives by highlighting the best time to provide additional support [7]. The potential for its application is enormous. Professor Mick Dodson of the Australian National University referred to data arising from The Longitudinal Study of Indigenous Children (LSIC) as “*points of light*” that join up and create a universe evidencing the successes of Aboriginal and Torres Strait Islander children [8]. Such high quality cohort studies have the power to demonstrate which factors, events and exposures in early life give these children the best chance to grow up strong and lead a healthy happy life.

Aboriginal and Torres Strait Islanders are regarded as the world's most researched people [9, 10]. A recent systematic review found that nearly half of all the longitudinal studies that focused on the health and well-being of young Indigenous children globally were found in Australia (41.9%, *n* = 88) [11]. The term ‘Aboriginal’ is used hereafter. While we acknowledge that this term does not include Torres Strait Islander peoples, it best represents the Central Australian population, and Aboriginal people in Central Australia have expressed their preference to identify in this way [12]. The review identified only three prospective cohort studies that included recruitment of local Aboriginal children from *Mparntwe* (Alice Springs) and the surrounding areas, but none were specifically designed to capture the unique experience of growing up in Central Australia. This lack of comprehensive longitudinal data on the healthy development of Aboriginal children in Central Australia, makes it difficult for the community to advocate for programs that are responsive to local context, culture, priorities and needs.

Past research efforts have not always benefited Aboriginal communities, an experience shared among Indigenous populations in other colonised countries. Decisions about what issues were to be addressed by research were often made in absence of community input. As a result, communities continue to be weary, and even cynical, of the value of research that is ‘investigator driven’ [9]. Aboriginal communities consider this type of research to be an extension of colonialism, benefitting researchers rather than communities [13, 14]. Moreover, biomedical-focused health research ignores Aboriginal knowledge systems that see health as more than just physical health but the social, emotional, cultural, and spiritual wellbeing of the whole community [15]. Reform has seen a demand for Aboriginal control over research in order to ensure Aboriginal priorities are met [13]. Best practice sees the identification of research priorities in partnership with community stakeholders. Project level proposals are now typically led by, or developed in partnership with, Aboriginal researchers and include ongoing guidance from community advisory and governance groups who have knowledge and expertise about local context.

The Central Australian Aboriginal Congress (Congress) in Alice Springs articulated the need for more research to focus on the health and wellbeing of young Aboriginal children as they grow during a research planning meeting in 2013. This later prompted talks of two partnership proposals between Congress and a group of researchers from the Baker Heart and Diabetes Institute, including senior Aboriginal epidemiologist SE [16]. The first proposal focused on the health and wellbeing of local Aboriginal adolescents and young people from 10–24 years of age whilst the second focused on the early life period from 0–5 years of age. The proposals were devised to help inform the health service and its partners about pathways that lead to better health outcomes. The first proposal was developed into a successful NHMRC funding application in 2015, this body of work was titled the ‘Next Generation Youth Wellbeing Study’ [17]. Following the success of the youth study, SE reconnected with partners at Congress to see if the service still sought longitudinal data on younger Aboriginal children as a research priority. Now affiliated with the University of Melbourne (UoM) and the Murdoch Children's Research Institute (MCRI), SE and a new team of researchers begun working in partnership with Congress to investigate the acceptability and feasibility of establishing an early life cohort study in Alice Springs.

It was felt that such a large-scale undertaking would need strong leadership from local Aboriginal researchers and key Aboriginal community groups and their members. Moreover, the direction and content of the future cohort study would need to be carefully co-designed and governed by a diverse group of local Aboriginal representatives to ensure that it remained responsive to local context, culture, priorities and needs. Similar cohorts of Aboriginal children have involved the collection of a variety of health, education and lifestyle information gathered using multiple data collection methods including direct face-to-face assessments and the low burden use of existing administrative data. These aspects would be decided by the community at a future time. Before this idea could progress, we asked the community what issues they would like the future study to focus on to ensure its potential benefit to the children of Alice Springs.

The objective of this qualitative study was to explore the local community’s priorities for young Aboriginal children and their families, so that the findings could be translated into research themes for a future proposed longitudinal cohort study in Alice Springs. This paper presents findings from the first phase of the *Atyepe-atyepe Iwerre Ampe-ke* [Healthy Journey for Kids] Feasibility Study conducted as a partnership project between Congress and MCRI.

## Methods

Participants were either parents or caregivers of young Aboriginal children (0–12 years of age) living in Central Australia, or community stakeholders with experience supporting young Aboriginal families in health, education, and community development settings. All were residents of Central Australia, at least 18 years of age, able to speak English, and provided consent to have their interviews recorded.

Potential participants were identified through a combination of purposive sampling and snowball sampling. We recruited within a 100-km radius of the township of Alice Springs, as serviced by Congress. To achieve maximum diversity, we purposefully sampled stakeholders based on their different roles across the health service, early childhood and education sectors [18, 19]. After interviewing a number of key stakeholders, we asked them to help identify other potential participants. The completeness of our approach was verified by the Research Manager at Congress who made initial contact with nominated stakeholders. An Information Statement & Consent Form was provided to all potential participants with an open invitation to discuss the study with a team member. Numerous opportunities to talk about the study with researchers were offered during the consent process. Once written informed consent was obtained an interview time and location were agreed upon. We were not able to conduct all of the interviews face-to-face due to the COVID-19 pandemic, some were conducted via video conference. In scheduling interviews, it was essential to be flexible in order to accommodate any social or cultural obligations of Aboriginal participants. Parents and caregivers were recruited most successfully through word of mouth and pre-existing networks. In close-knit Aboriginal communities, the 'snowball' approach to sampling has proven effective and culturally accepted [20].

Interviews with parents and caregivers were conducted in-person by either AH, ES or SM. Our initial aim was to include between 15 and 30 participants in total. Other qualitative studies looking to identify patterned meaning across data have used a similar sample size range [21]. Recruitment was extended until data adequacy was reached (richness/ complexity) to tell a multi-faceted story [22]. The interview guides were informed by the literature on priority setting in health research involving Aboriginal and Torres Strait Islander communities. Open-ended questions were adopted so that participants could talk freely about their perspectives on health priorities for young Aboriginal children in Alice Springs (Additional file 1). We started off each conversation asking participants to tell us what they thought local Aboriginal children needed to have the best start in life. This then led to discussion about which specific issues at different milestones as kids grow, and how the proposed cohort study should address these issues. Questions were adapted slightly to best suit each individual participant. The first author (CLJ) developed the first iteration of each guide in consultation with the lead Aboriginal Investigator (SE). Further refinement was conducted by all authors following pilot testing with Aboriginal and Non-Aboriginal peers, both of whom were experienced qualitative researchers. Staff involved in the collection of data were familiar with the guides and had prior qualitative research experience. Voice recorders were used to digitally record interviews and FGDs conducted in person in Alice Springs and via video conference. Both verbal and non-verbal language were monitored throughout. Questions were asked differently if the interviewer or facilitator felt that any of the participants were disengaged. Participants' social and emotional safety were closely monitored as per protocol. No signs of distress were detected. Each question was followed by a period of silence so the participant could think about the question before responding, which is considered a culturally responsive practice [12]. As advised by the Lead Female Cultural Advisor at Congress, care was taken to ensure culturally based gender sensitivities were considered when planning each interview or FGD [12]. Two Aboriginal female researchers (AH & ES), a male non-Aboriginal researcher (SM) & a female non-Aboriginal researcher (CLJ) conducted data collection. Each FGD began with an explanation of its purpose and structure, as well as an emphasis on confidentiality. While ensuring topics were covered according to the FGD guide, facilitators (AH & SM) allowed discussions to flow naturally. In order for participants to focus on their own experiences, prompts were provided. All Aboriginal participants declined the offer of an interpreter and elected to be interviewed in English. As a thank you for taking part, participants received a $20 gift card. Data was collected over a 14-month period from 2020 to 2021.

Reflexive thematic analysis (TA) was conducted following Braun & Clarke's (2006) six-step approach [23, 24]. The audio recordings were manually transcribed verbatim and cross-checked for accuracy by SM & CLJ. Anonymized transcripts were then imported into NVivo qualitative data analysis software (Version 12, 2012, QSR; International Pty Ltd. Melbourne, Australia). To become familiar with the data, CLJ and SM independently read and reread each transcript. Brief memos and reflections were drafted. The first author (CLJ) led Phase 2 of the data analysis coding text segments in each transcript into preliminary nodes using a bottom-up approach**.** Both semantic and latent features of the data were examined. At least one transcript was independently reviewed by AH and ES, and SM coded 10 transcripts. This exercise was not intended to test inter-rater reliability or reach consensus. Instead, we wanted to create a collaborative interpretation of the data in a space where we compared and reflected on our different perspectives as a team. In doing so, we were able to gain a deeper understanding of the data [25]. The bottom-up approach we used to code allowed us to formulate themes based on the data rather than predefining them. A number of finely grained themes emerged from the first iteration of nodes (Phase 3). All transcripts coded independently by the team were reviewed by CLJ to determine whether any other relevant themes needed to be added. After reading all the data under each node, SM, AH, and ES discussed the preliminary themes CLJ had identified. Through reflexive TA, the authors acknowledged their active role in generating new knowledge. It was important for the authors to discuss how their experiences and positions might impact their interpretation of the data (refer to Positionality Statement in Additional file 2). The team met to share their understanding of the data and the preliminary themes. Phase 4 involved refining and synthesising the candidate themes from four to three major themes with several sub-themes each. It was agreed that the final theme names conveyed a shared meaning across the dataset (Phase 5).

**Fig. 1:**
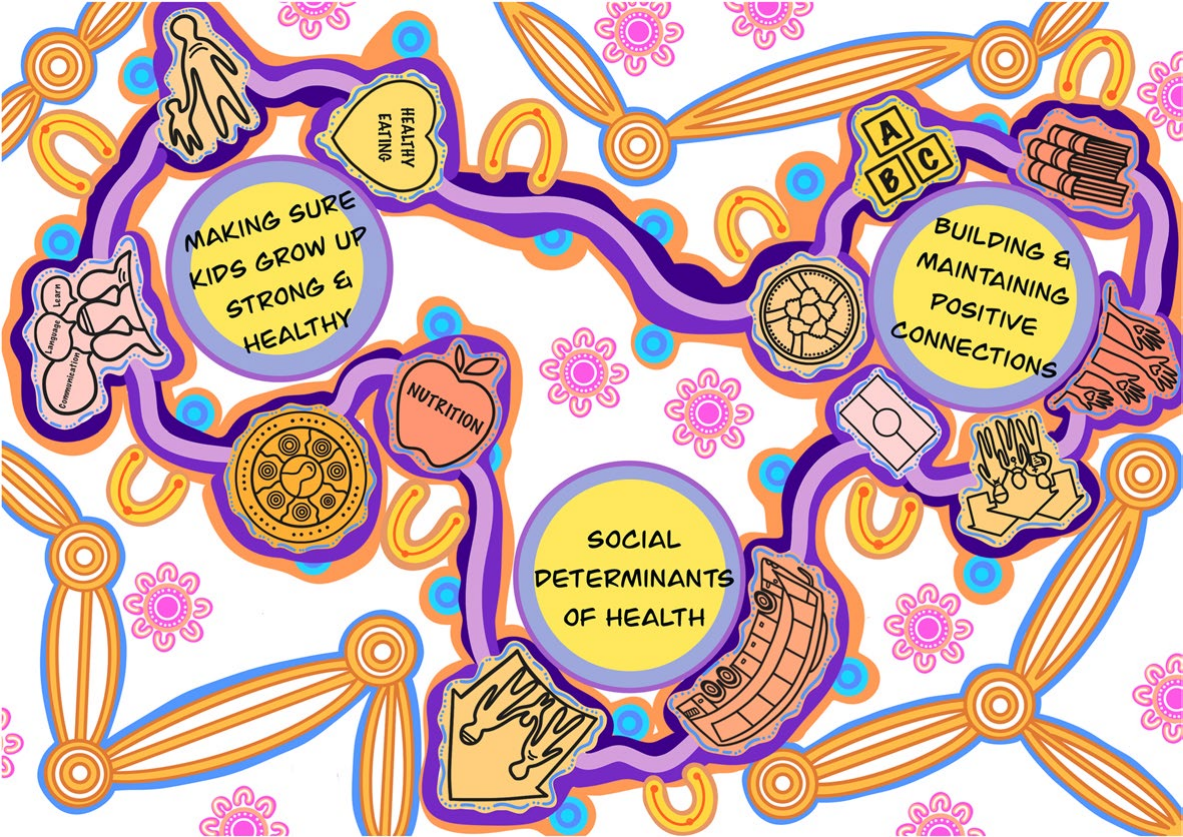
Thematic mind map

The research is positioned within a social constructionist perspective, which affirms that people interpret and make sense of their experiences based on their social, political, and historical contexts [26]. Themes and sub-themes were further refined during the writing process in Phase 6 (refer to Fig. 1). A strengths-based approach was adopted in framing the final results [27]. Our research is also situated within a transformative paradigm, which assumes that knowledge reflects power relations in society, and strives to redress this situation by privileging the voices and needs of the local community [14].

The final manuscript was written by all authors and approved for publication by Congress prior to peer review. The three Aboriginal researchers (AH, ES, and SE) provided cultural guidance on the presentation of findings. COREQ guidelines were followed in the conduct and reporting of the study (Additional file 3). The project was endorsed by the Congress Research Sub-Committee prior to receiving formal ethics approval from the Royal Children’s Hospital Human Ethics Research Committee (2019.155) and the Central Australian Human Research Ethics Committee (CA-19–3519) in 2019. The Congress Research Sub-Committee includes Aboriginal leadership and exists to support and promote research that reflects and is responsive to the needs of the local Aboriginal community. Permission was also granted by the NT Department of Education to engage with local primary school educators. We conducted this study in accordance with the ethical guidelines for best practice in research involving Aboriginal and Torres Strait Islander communities [12, 28–36].

## Results

During the period of August 2020 to October 2021, 27 interviews and three FGDs were conducted with 42 participants. There were between three and five participants in each FGD, which averaged 83 min (range 65—97 min). Each interview lasted about 41 min (range 14—68 min). The study included 36 women and six men, ranging in age and occupational status (Table 1). A total of 16 parents or caregivers of young Aboriginal children and 26 community stakeholders were included in the sample. Stakeholders were directly involved in providing education, healthcare, and/or community services to local Aboriginal families. Among them were Aboriginal and non-Aboriginal health workers, doctors, allied health professionals (*n* = 7), teachers (*n* = 7) or early childhood educators (*n* = 5), and other community service providers (*n* = 7). Over half of the sample (57%) self-identified as Aboriginal. Aboriginal stakeholders spoke of their dual roles as community members and caregivers for their own children, grandchildren, and/or extended family members. Some participants spoke of family connections reaching as far as South Australia, Western Australia, and Queensland in addition to Central Australia and the Northern Territory. Many of the Aboriginal participants were speakers of one or more Aboriginal languages. To maintain anonymity, we have removed identifying characteristics from participant quotes.

**Table 1:** Participant characteristics

**Participant characteristics**		
	**Number**	**Percentage (%)**
**Gender**		
Female	36	85.7
Male	6	14.3
**Aboriginal**		
Yes	24	57.1
No	18	42.9
**Role**		
Parent/Caregiver	16	38.1
Stakeholder/ Community representative	26	61.9

Here we present the results of our reflexive thematic analysis (Fig. 1). Three overarching themes as well as subthemes were identified from participant responses: 1) social determinants of health (2) building & maintaining positive connections, and (3) making sure kids grow up strong and healthy. These interconnected themes illustrate research priorities and areas of concerns participants wanted future research to focus on.

### Theme 1: social determinants of health

All of the participants expressed a shared sense of responsibility and purpose to improve the health and well-being of local Aboriginal children. We found a general positive attitude toward the idea of collecting longitudinal data on local children as reported elsewhere [37]. The priority issue most frequently discussed related to the underlying social determinants of health (SDH) that have enormous influence on health inequities experienced by the community. SDH are “the conditions in which we are born, grow, live, work and age” [38, 39]. These determinants include, among others, income, housing, education, food, our access to and use of health services. Together, these conditions provide people with the freedom to live the lives they choose [40]. For Aboriginal people, the SDH also includes culture, language, family and kinship, and access to traditional homelands. These factors are interrelated, complex and are shaped by the distribution of money, power and resources at all levels [3]. Respondents overwhelmingly favoured research that would look into how these factors shape children’s health and development trajectories:

*“All the social determinants of health: housing, lack of opportunity, (not) having access to services… (that’s what) prevents our families fixing their health” (Female, Aboriginal Primary Health Care Worker)*

One stakeholder emphasised the SDH as being an important issue to address:

*“I think in capital letters, 72 font, bold, underlined, and in italics, [that the] social determinants of health are the number one driver of… excess health burden in our population of children” (Female, Non-Aboriginal Primary Health Care Professional).*

The cycle of poverty and disadvantage was noted by many respondents as a common experience for Aboriginal families in Central Australia:

*“Its everyday life… everyday struggles... You know? (For some families) not having a house to go home to… No food. It's all that (kind of) struggling (that impacts kids)” (Female, Aboriginal Parent/Caregiver).*

To make an impact, it was considered important that the future study aim to better understand these underlying causes affecting children’s security and stability as they grow. The underlying impact of poverty and intergenerational trauma was often cited as needing attention. The Aboriginal understanding of a child’s health is that it is intricately connected to the health of the whole community. This was clearly articulated across the data, participants advocated that the future study should also embrace this holistic understanding of health in its approach to data collection:

*“The study should focus on… the child as a whole… We need to know the child as a whole and everything that's affecting the child has to be looked at... That's the only way” (Female, Non-Aboriginal Early Childhood Educator).*

Timing was also identified as important, many recommended that the future study should focus on children in the early life period when there is greatest opportunity for change. A few important nuances in the types of issues raises by participants that warrant further reflection. We found health practitioners were more likely to talk of specific biomedical conditions and health conditions as opposed to the broader SDH, such as iron deficiency and growth faltering. As expected, educators focused more on the importance of access and engagement in education, though they too frequently returned to how the broader societal issues impacting families complicated their best efforts.

#### Subtheme (a) Adequate housing

One of the many basic SDH is access to adequate housing. This stood out as one of the biggest factors affecting the security and stability of local Aboriginal children and their families in Alice Springs. The absence of safe, secure homes with working facilities was identified by all participants as a key determinant of persisting inequities for young families warranting further investigation. Health care professionals identified the strong links between insufficient housing or overcrowding and ill health, including but not limited to recurrent bouts of pneumonia, gastroenteritis, chronic scabies and skin infections and rheumatic heart disease. Whilst others talked of the causes, including how periodic mobility within town, and between town and homelands, was a common contributing factor to overcrowding. Several parents and caregivers noted how cultural obligations to extended family and kin made preventing overcrowding a difficult issue to tackle:

*“If you're from…Central Australia [you have] so many different families coming to visit and... it's hard to say no” (Female, Aboriginal Parent/Caregiver).*

Concerns about insecure housing were prevalent among the sample. Several participants suggested that research should look into the long term trajectory of children experiencing housing instability and homelessness.

#### Subtheme (b) Access to transport

Another priority issue referenced across all of the interviews was the need for reliable transportation for young families. Transport was reported as a key enabler for children to access school and health care services. This was recounted as especially problematic for school aged children:

- *“Access to education… in remote communities outside of Alice Springs is definitely a transport issue” (Female, Aboriginal Primary Health Care Worker)*
- *“Years ago, they used to have buses connected with all the primary schools and they would go around and pick up vulnerable kids and take them to school. Well, they don't now… they have to catch public transport or [rely on their] parents” (Female, Non-Aboriginal Primary Health Worker).*

Several respondents linked the defunding of school transport programs to lower attendance levels. Taking public transport was not something parents always felt comfortable sending their young kids for fear of bullying and racism on the bus. One stakeholder reflected that; in her experience the easier option was that kids don’t go to school*.* This was described as a major issue, particularly for children living on outstations or remote communities surrounding Alice Springs. It’s not only access to a reliable vehicle that is the issue but the running costs too. A couple of parents and caregivers, in particular, spoke of the negative impact on children’s ability to participate in sport or other extracurricular activities. The need to better understand the links between transport accessibility and well-being for local children over time was highlighted as important to the community.

### Theme 2: building & maintaining positive connections

Another important theme identified was the need for research to focus on positive connections for children that give them the best start to life.

#### Subtheme (a) Positive parenting & attachment

All participants expressed a strong desire for local children to feel loved and valued, to grow up in a safe environment surrounded by people who genuinely care about them and help them establish good routines to thrive:

*“The biggest issue I'd say…is ensuring that [they]… have a safe home environment… where there's a routine, a structure… [where their] …basic needs [are] being met. [Like] hygiene, …food, going to school… If you don't have it early on then… a lot of children will find it difficult” (Female, Aboriginal Parent/Caregiver).*

Participants shared a view that children need at least one parent (or caregiver) that is present and engaged early in life to have “a strong sense of attachment”. Positive early connections with parents, caregivers and extended family networks were considered integral:

*“The most positive thing in their life, the strongest protective factor for Aboriginal kids is their families, good, strong, healthy, happy families…All kids need to feel attached and loved… that's what builds emotional stability and capacity… and a good start [to life]” (Female, Non-Aboriginal Health & Education Officer).*

Participants want future research to focus on the many strengths of Aboriginal family functioning and not view differences from non-Aboriginal family structures and practices as deficits. Stakeholders, in particular, were interested in the long term impact of parenting and family support programs in laying the foundation for future positive social and emotional development.

#### Subtheme (b) Connection to culture

Other connections participants referred to include a child’s need to establish and maintain connections to culture, language and Country through their immediate family, extended kin and the broader community. These aspects were highlighted by many participants as offering spiritual support, a sense of identity, belonging and pride:

*“I want to see them thrive… and speak up when they need support… and be strong within themselves, their identity, their culture, their community” (Female, Aboriginal Parent/Caregiver).*

A best start to life for many interviewed represented “*growing them up strong in culture*”. This was described by one Grandmother as essential to prepare them for their future, giving them knowledge of how to live in “two worlds”. Hence, connected communities contribute to their holistic health and well-being. It was recommended that future research should include exploring culture as a protective factor for children as they grow*.* Greater recognition of the complex challenges faced by young Aboriginal children and their families navigating ‘two worlds’ was also expressed as a priority.

#### Subtheme (c) Access & engagement with education services

Education was expressed by all as a critical SDH by all respondents. Education is closely related to other SDH that influence experience of positive health outcomes across the life course, beginning in childhood [3]. Connections children develop over time with education and health services was expressed as an important issue warranting further attention in research. Participants acknowledged the many challenges experienced by families to remain engaged with in the education system including past negative experiences. Several practical reasons why some families find it hard to get their kids to school were referenced in the data, including the mobility of some families within and between communities and the lack of transport. The struggles some families face with day-to-day survival was also acknowledged as impacting parent’s ability to get their children to attend school regularly. Concerns were raised about the long term impact for children who do not establish positive connections and engagement in schooling from an early age. The transition period from preschool to school was identified as an important timepoint that needs specific attention. One grandmother believed that this time is especially important for local kids:

*“Early intervention is [a] really good one… cause you can… target what kids need... before they go to preschool… Education starts at home…actually… to get that kid to go to school and everything. Because if you don't have… parent[s] that… push…and guide that child to school…. [then] they just fall of the rails” (Female, Aboriginal Parent/Caregiver).*

Overall, participants talked of the need to focus on building positive connections with educators and the education system to give Aboriginal children the best start to their educational journey. There was a common view that schools need to be culturally responsive and inclusive places. One of the stakeholders talked of how the education system should really be adaptive to the specific needs of local Aboriginal children:

*“I want to see young Aboriginal children… engaged in an education system that actually fits them... they need to be offered schooling systems that fits them and their families… These kids need their language…[and] culture privileged… Our education system is broken as it is… I think, it starts with education, and I know that it starts with the education of mums because that's where the world starts to change” (Female, Aboriginal Primary Health Worker).*

Many of the educators interviewed talked of their schools being more than just a bricks and mortar place to learn, reflecting that for some children it is their “*safe place”.* We asked each participant about their hopes and dreams for young Aboriginal children in Alice Springs. Parents and caregivers wanted greater opportunities for their children than they had themselves and hoped that their children could reach their potential and achieve their goals in life. All responses included reference to the need for good education and timely intervention in order to achieve the best start in life.

#### Subtheme (d) Access & engagement with health services

Building positive connections with health services was another sub-theme. Such connections were noted to be complicated by various factors like access to allied health professionals and/or specialists in town. The SDH was again highlighted as impacting families’ ability to sustain these connections with health services. One of the stakeholders talked about families who struggle to engage with services that are not culturally responsive. Another issue that arose was the need to better understand why some families refuse to engage with specific services or received treatment or diagnosis. It was suggested that some families are fearful of certain outcomes that may reflect poorly on them:

*“We have one family right now… who has clearly said… that she doesn't wish to engage with us as a diagnostic service for fear of the outcome, but more so the inclusion of child protection… fearing that the diagnosis is going to reflect on her in some way and therefore child protection steps in. So, it's a real fear and that's absolutely an issue, I think [that] needs exploring” (Female, Non-Aboriginal Primary Health Worker).*

Several stakeholders thought it was important for future research to look at what services and support (including screening and intervention) families engaged with over time and see what sort of impact these have had for children.

### Theme 3: making sure kids grow up strong and healthy

The third theme identified revolves around a few specific issues frequently raised as a priority for local Aboriginal kids to grow up strong and healthy.

#### Subtheme (a) Healthy mum & bub

When discussing the spectrum of specific health issues affecting local Aboriginal children, many participants kept returning to how it all stems from the health of the mother before birth, during pregnancy and the first 1000 days of life. One of the stakeholders commented that:

*“I think it's clear that… [a child’s] journey starts in utero, and so looking after mums… and making sure [they] have the information they need for little babies to get that strong start [is really important]” (Female, Non-Aboriginal Allied Health Professional).*

The focus of any future research was expressed as needing to include examination of these important periods of health and development.

#### Subtheme (b) Language, communication & capacity to learn

Another reoccurring issue raised by interviewees centred around language, communication and children’s capacity to learn. Both parents and stakeholders talked about the high rates of ear disease, hearing loss and speech delay:

*“Ear issues…. affect their listening skills, their communication, their social skills, [their ability] to meet milestones because if you can't hear... you can't socialize, you can't talk…You know, it's really hard. Even your social skills are not developed because you can't hear what others are saying” (Female, Non-Aboriginal Early Childhood Educator).*

Many local children speak one or more Aboriginal languages in addition to English. Speaking an Aboriginal language is a great strength, but a few participants admitted that this can present additional challenges for Aboriginal kids when they enter formal education which is largely taught in English:

*“We see a lot of kids who…come from families where English is not their first language… [so] we are concerned for those kids who have not yet developed enough of those basic English skills” (Female, Non-Aboriginal Primary Health Worker).*

Investigating the complex and interconnected factors that impact Aboriginal children’s early language and literacy development, and their capacity to learn, was seen by all participants as a valuable priority issue to pursue.

#### Subtheme (c) Nutrition & healthy eating

There were strong feelings that future research should also include a focus on nutrition and healthy eating. Parents and stakeholders were concerned with the rising number of children and adolescents diagnosed with Type 2 diabetes. Promoting healthy eating and having access to high quality fresh produce were discussed as priorities for local children. Looking into the multifactorial issues contributing to food insecurity was also important. Educators in particular brought this up as a “big issue” saying:

*“a lot of kids come [to school] with no food and they're tired… they end up having to have a sleep in the middle [of the day]. There's so many… in my class…they're hungry as soon as they get here” (Female, Non-Aboriginal Primary School Educator).*

Undernutrition and growth faltering were also identified as areas of concern. Several health professionals interviewed referred to the high rates of nutritional deficiencies and babies born premature or small for gestational age*.*

#### Subtheme (d) Social & emotional wellbeing

Different aspects of children’s social and emotional wellbeing were discussed in the interviews. Whilst the majority of Aboriginal children grow up in stable home environments, and live in safe communities, there are some that do not [41]. Participants acknowledged that some families find it difficult to overcome trauma from past events. This trauma can pass to children (inter-generational trauma) and is often compounded by family violence and drug and/or alcohol misuse [41].

One participant suggested that local children and young people “*need role models*” and another said that “*they need to [support to re]-connect with family*”. Participants were united in their concern for the long term impact of children who experience trauma. Educators in particular, asked for more research to focus on how they can better support children who experience trauma:

*“Children who face trauma… [are] coming into classrooms [and] are not coping... [they are] hiding under blankets and [are] frightened…. We're having to do things to support them... and have a safe place for them. School is a safe place for a lot of them” (Female, Non-Aboriginal Primary School Educator).*

Another aspect of children’s SEWB raised in the interviews related to racism and discrimination:

*“There is a lot more racism in town…. [Like] not being allowed in a building, being followed, or watched. Kids see all of that… all the time and that sends a huge message to kids about their connectedness to community, and which community they're connected to, and where they're not welcome” (Female, Non-Aboriginal Allied Health Professional).*

Exposure to these pervasive stressors can be devasting and have long term impacts and warrants further investigation. Having a community that fosters their confidence instead of looking at deficits was also deemed of critical importance to respondents.

## Discussion

This study sought to identify research priorities for local Aboriginal children in Alice Springs. Thematic analysis of qualitative data from interviews and FGDs with 42 participants (57% Aboriginal identified) generated three broad themes. The first theme emphasises the need to better understand the complex and interrelated SDH from a holistic concept of health. Key informants explained in confident, authoritative terms how issues affecting young Aboriginal children are interconnected with the SDH and the historical legacy of colonisation. The expressed need for children to form strong connections, especially with their primary caregivers, shaped the second theme. Several specific health issues were raised as important to the community in the third theme. This included nutrition and healthy eating, ear disease and hearing loss, language and literacy development, children’s capacity to learn and their SEWB. Differences between parents and stakeholders were not unexpected. In general, parents were more concerned about broader public health issues and educational attainment rather than specific diseases. Stakeholder type influenced specific issues reported as important, for example educators were concerned about school readiness, attendance and food security affecting children’s ability to concentrate in class [42], whereas medical professionals expressed concerns about growth faltering, iron deficiency, and recurrent infections caused by overcrowding.

A child’s trajectory or journey from birth to adulthood is best captured by longitudinal research. Respondents saw the value in the proposed longitudinal cohort study. We asked participants if there were certain topics or issues that should be considered taboo or “off limits” and the general consensus was “No”. However, sensitive topics need to be negotiated carefully using a trauma informed lens with leadership and guidance from local Aboriginal peoples. Greater concerns were raised by participants about future unnecessary duplication of data that might add burden on families. The acceptability of the proposed longitudinal cohort study has been discussed in more detail elsewhere [37]. In brief, the future study needs to be developed with strong local Aboriginal leadership and governance and framed from a strengths-based approach.

This research has both strengths and limitations. An important strength of this study is that it was led by Aboriginal researchers (SE, AH & ES) and conducted in partnership with an Aboriginal controlled health service. AH & ES were able to draw on their personal networks, knowledge and experience to provide a culturally safe space for participants to describe what issues were most important to them. Our combined contribution to the thematic analysis ensured that we were able to integrate multiple perspectives and positions in the interpretation of the data.

We acknowledge that our study focused on the township of Alice Springs and its immediate surrounding areas. Families living in town camps were not specifically targeted in this sample. However, some of the caregivers identified through snowball sampling were previous town camp residents. The majority of our participants were adult females living in urban areas around the township of Alice Springs. Overall, male participants were underrepresented in this study (*n* = 6). All stakeholders were female. This is consistent with the high number of female workers in the Australian health care, social assistance, and education sectors [43]. A deliberate effort was made to ensure that Aboriginal voices made up more than half of the sample (57%). Our results should be interpreted in this context. It is unknown how well these findings generalize to other geographical locations; however, we believe some of the issues identified may be relevant to other remote Indigenous communities with a shared colonial history.

Of note, our timelines were impacted by the COVID-19 pandemic. The NT border was closed for extended periods of time during 2020/21 impacting the ability of Victorian based author (CLJ) to conduct fieldwork locally. Anyone entering the NT at the time was required to stay in hotel quarantine for two weeks, and remote communities were closed to all non-essential visitors. In order to overcome these disruptions to our schedule, we decided to extend the data collection period. The first author (CLJ) pivoted to collect interview data from several stakeholders remotely using a video conferencing platform. All interviews with parents and caregivers were conducted in person by local authors (AH, ES & SM) following COVID-safe protocols. We obtained more than 32 hours of audio-recorded data in spite of these limitations from a broad range of participants.

## Conclusion

Participants believed that future research should first and foremost address some of the underlying SDH, in particular issues around housing, overcrowding, socio-economic disadvantage and access to transport. In addition to the SDH, the community wants focus placed on children’s learning and general development, family and community connections, and a select few health topics including ear health, nutrition and SEWB. These research priorities provide a preliminary roadmap for the next phase which will involve further community engagement to co-design the study protocol with local Aboriginal leadership and governance. Based on our findings, we advocate for the adoption of a holistic concept of health and well-being that showcases the many strengths of the local community, including the protective factors of culture, language and connection to Country. Our findings represent a challenge for the co-design team who will now be tasked with developing a study protocol that meaningfully operationalises the SDH and how it could be measured overtime whilst ensuring low impost on local families to produce answers to some of the priorities identified as important to them.

### Supplementary Information

**Additional file 1.**

**Additional file 2.**

**Additional file 3.**

**Additional file 4.**

## Data Availability

Data are available upon reasonable request and with permission of the Central Australian Aboriginal Congress Research Sub-Committee via the corresponding author.
