# Supplementary material for: “I want to see them thrive!”: exploring health service research priorities for young Aboriginal children growing up in Alice Springs – a qualitative study
Source: BMC Health Serv Res. 2024 Feb 15;24:205. doi: 10.1186/s12913-024-10642-8 (PMC10868103; doi:10.1186/s12913-024-10642-8)
Supplement: Supplementary file 1 — Additional file 1. [file 12913_2024_10642_MOESM1_ESM.docx]

# **ADDITIONAL FILE 1**

**“I want to see them thrive!”: exploring health service research priorities for young Aboriginal children growing up in Alice Springs – a qualitative study”**

**SEMI-STRUCTURED INTERVIEW GUIDE**

| WELCOME, OVERVIEW & INFORMED CONSENT |
| --- |
| - Thank participant for agreeing to take part. - Re-introduce yourself - Explain that the interview will go for up to 90 minutes. - Stress there are no right or wrong answers - Explain that the interview will be guided by a series of open-ended questions - Explain that they can choose whether to answer the questions or not at all - Explain that they can take a break or stop at any point during the conversation if they become uncomfortable - Explain that we are not going to use jargon but if we do, we will explain what we mean - Reassure them of confidentiality and anonymity - Go over the plain language statement & describe the aims of the research - Gain informed consent - Ask for permission to record – for analysis purposes only   *Do you have any further questions before we start?... Ok I’ll start recording now*  TURN ON RECORDER |
| WARM UP & INTRODUCTIONS |
| - Ask participant to briefly introduce themselves: - Name & age group - Gender - Cultural background (identify as Aboriginal, and/or Torres Strait Islander?) - Postcode (suburb of residence)   (Parents/caregivers)   - What languages do you speak at home? - How many kids do you care for? - What are their ages?   (Stakeholders)   - Tell us a bit about the work you do in supporting Aboriginal families? |
| HEALTH OF YOUNG ABORIGINAL CHILDREN IN ALICE SPRINGS |
| *The first set of questions I’ll ask are about local health needs and priorities for young Aboriginal children in Central Australia.*   - What do you think Aboriginal children need to have the best start in life (so they can grow up strong, healthy & do well in life)? - What do you think counts as a ‘best start’? - What things might stop kids from growing up strong/healthy? Ok, what about things that helps them stay on track? - What are your hopes and dreams for young Aboriginal children growing up in Central Australia? - What do you think are the most important issues for young kids in your community (in general)? - What about babies & toddlers (0-2 years)? - What about for preschool aged kids (3-5 years)? - And, for primary school aged kids (6 to 12 years)? - Is there anything else you would like to comment on or mention (about the health of young Aboriginal children)? |
| LONGITUDINAL RESEARCH |
| *Ok…the next questions are about your views on research in general*  (Parents/caregivers)   - Can you tell me if you have ever taken part in a research study before? - Tell me about your experiences? - Has anyone in your family taken part in a research study before?   (Stakeholders)   - Can you tell me if your organization collects and/or uses data on the health and well-being of Aboriginal children? - How are the data collected? How often are the data collected? How recent are the data? - What do you think are the current data gaps? - Are there any problems with the data?   *I’m going to show you <flip chart or picture> and explain what a cohort study is … Then I’d like to ask you for your thoughts/opinions specifically on the idea of setting up a local ‘cohort study’*  *Show participant flip chart or PICTURE and provide explanation*   - Do you have any questions? - What do you think about the overall idea for a local cohort study? - What are the things you like about it? - What are the things you don’t like about it? - How might this information be useful to you (& your family)? - How might it be useful to your community? - Do you believe it is needed or important? - What impact do you think it could have on local community child health and wellbeing programs? - Anything that didn’t make sense? - Any worries you have about this kind of research? |
| DATA LINKAGE |
| *Just like before I’m going to show you <a flip chart or picture> about what data linkage is and how this might be used in the proposed cohort study… it will only take a few minutes. Then I’d like to ask you for your thoughts/opinions on data linkage.*  *Show participant flip chart or PICTURE and provide explanation*   - Anything that didn’t make sense? - What are the things you like about it? - What are the things you don’t like about it? - Any worries about data linkage? |
| STUDY FOCUS |
| *Thinking back to what you said were the most important health issues for kids…*   - What do YOU think the study should focus on? - Can you think of anything the researchers should NOT do, or not include? Any topics that are off-limits? - Or any specific conditions or limitations? |
| ACCEPTABILITY |
| (Parents/caregivers)   - How would you feel about your [child/ niece/ nephew or grandchild] taking part in a study like this? - Can you tell me more about your decision (why/why not)? - What type of involvement/contact would you like to have with the researchers? - What about the type of contact? I.e., Face to face? Telephone? Mail? Email? Combination? - How about how often families should be contacted? I.e., Once off? Every 12 months? Every 24 months? Data Linkage only?   (All participants)   - What do you think might help or encourage families to take part? - What do you think might stop families from wanting to take part? - What would you want out of the study (either personally or professionally, for your own family and/or families in your community)? |
| CLOSING DISCUSSION |
| - Are there any issues that we haven’t discussed that you would like to discuss or comment on? - Explain that the interview is pretty much finished. - Explain that we will shortly send out a thank you card and that they will receive a summary of the findings in about 12 months’ time - Ask if they can suggest other individuals who we should consider talking to - Thank the interviewee for taking part   TURN OFF RECORDER  END OF INTERVIEW |

**FOCUS GROUP DISCUSSION GUIDE**

| TIME |  |  |  |  |
| --- | --- | --- | --- | --- |
|  | **WELCOME** | | |  |
|  | **Acknowledgment of Country** | | |  |
|  | **Overview & Informed Consent**   - Go over study objectives & PICF - Explain FGD will go for up to 2 hours - Refreshments available: please help yourselves - Can take a break or stop at any point during the conversation if you become uncomfortable or to use bathroom - Not going to use jargon but if we do, we will explain what we mean - Reassure: confidentiality and anonymity - Ask for permission to record – for analysis purposes only | | |  |
|  | **Ground rules**   - 1 person speaking at a time - There are no right or wrong answers - When you have something to say, please do so - You don’t have to agree with other people in the group. | | |  |
|  | **ICEBREAKER & INTRODUCTIONS** | | |  |
|  | **Show of hands;**   - How many people here are parents? - How many grandparents do we have? - What about aunties or uncles, caregivers, and guardians, how many do we have here today? - Do we have anyone who works with or supports Aboriginal families? | | |  |
|  | **Facilitator & RA introductions** | | |  |
|  | **Participant introductions:**   - Tell us you name, your age, and a bit about yourself?   (Parents/caregivers)   - What languages do you speak at home? - How many kids do you care for? - What are their ages?   (Stakeholders)   - Tell us a bit about the work you do in supporting Aboriginal families? | | |  |
|  | **GROUP DISCUSSION- PART 1^[[1]](#footnote-1)^** | | |  |
|  | - What do you think Aboriginal children need to have the best start in life (so they can grow up strong, healthy & do well in life)? - What do you think counts as a ‘best start’? - What things might stop kids from growing up strong/healthy? - Ok, what about things that helps them stay on track? - What are your hopes and dreams for young Aboriginal children growing up in Central Australia? - What do you think are the most important issues for young kids in your community (in general)? - What about babies & toddlers (0-2 years)? - What about for preschool aged kids (3-5 years)? - And, for primary school aged kids (6 to 12 years)? | | |  |
| *FACILITATOR: “Now we’re going to talk about data and research… relating to how we can follow the health of Alice kids… but first tell me…”* | | | |  |
|  | **Show of hands**   - Has anyone taken part in a research study before? - What about anyone in your family…? | | |  |
| *FACILITATOR: “I’ll explain a bit more about these things…” <explain life course/longitudinal research using flip chart/whiteboard>* | | | |  |
|  | - Has anyone heard of this kind of research (“longitudinal”)? - Anyone participated in something similar? - Anything that didn’t make sense? - Or does anyone have any questions? - What are the things you like about it? - What are the things you don’t like about it? - What do you think about the overall idea for a local cohort study? - Any worries you have about this kind of research? | | |  |
| *FACILITATOR: “Just like before I’m going to show you < flip chart or a picture> about what data linkage is and how this might be used in the proposed cohort study… it will only take a few minutes. Then I’d like to ask you for your thoughts/opinions on data linkage”.* | | | |  |
|  | - What do you think about ‘data linkage’? - Anything that didn’t make sense? - Or does anyone have any questions? - What are the things you like about it? - What are the things you don’t like about it? - Any worries about data linkage? - What do you think might help or encourage families to take part? - What do you think might stop families from wanting to take part? | | |  |
| *FACILITATOR: “Ok, let’s have a short 10-minute bathroom break. Please feel free to grab another cuppa & meet back in circle in 5 minutes. When we come back, we are going to talk about ______________”.* | | | |  |
| 10 MIN | **BREAK** | | |  |
|  | *(If required)* | | |  |
|  | **GROUP DISCUSSION- PART 2** | | |  |
|  | - What do you think might stop families from wanting to take part? - How would you feel about your [child/ niece/ nephew or grandchild] taking part in a study like this? - Would you be happy with them taking part? Or be hesitant? - Tell us a bit about your decision? - Ok, for those who said “Yes” they would be ok with it …. What type of involvement/contact would you like to have with the researchers? - What kind of contact would be ok with you? - None (data linkage only)? Face-to-face? Telephone? Mail? Email? Combination? - How often would be happy to be contacted? - Once off? Every 12 months? Every 24 months? Data Linkage only? - Thinking back to what you said were the most important health issues for kids… What do YOU think the study should focus on? - Is there anything the researchers should NOT do, or not include? - Any topics that are off-limits? Or any specific conditions or limitations? - Do you think it could be useful? - To you and your family? To your job/organisation? To the community? - Do you think having this ‘data resource’ is needed or important? - How do you think the data should be managed/looked after over time? - What kind of specific conditions or limitations should there be? - How would you like the data/results be shared with community? | | |  |
|  | **CLOSING DISCUSSION** | | |  |
|  | - Are there any issues that we haven’t discussed that you would like to discuss or comment on? | | |  |
| 2 MIN | **CLOSING REMARKS** | | |  |
| *FACILITATOR: Thank you for taking part <today/this evening>. It’s been a really successful discussion and your opinions and comments will be a great help to us. We hope you found it interesting. We will be giving everyone a summary of the findings from this phase of the project in about 12 months’ time. Once again thank you all for coming <today/tonight>!* | | | | |

1. Should any participant (including pregnant women) become uncomfortable or distressed during the focus group discussion- research assistants are required to follow the steps listed in Section 5 of the Study Protocol as per training. [↑](#footnote-ref-1)
