# Supplementary material for: “I want to see them thrive!”: exploring health service research priorities for young Aboriginal children growing up in Alice Springs – a qualitative study
Source: BMC Health Serv Res. 2024 Feb 15;24:205. doi: 10.1186/s12913-024-10642-8 (PMC10868103; doi:10.1186/s12913-024-10642-8)
Supplement: Supplementary file 3 — Additional file 3. [file 12913_2024_10642_MOESM3_ESM.docx]

# **ADDITIONAL FILE 3**

**“I want to see them thrive!”: exploring health service research priorities for young Aboriginal children growing up in Alice Springs – a qualitative study”**

**COREQ (COnsolidated criteria for REporting Qualitative research) Checklist**

Developed from: Tong A, Sainsbury P, Craig J. Consolidated criteria for reporting qualitative research (COREQ): a 32-item checklist for interviews and focus groups. International Journal for Quality in Health Care. 2007. Volume 19, Number 6: pp. 349 – 357.

| Topic | Item No. | Guide Questions/Description | Reported on  Page No. |  |
| --- | --- | --- | --- | --- |
| Domain 1: Research team and reflexivity | | | |  |
| Personal characteristics | | | |  |
| Interviewer/facilitator | 1 | Which author/s conducted the interview or focus group? | Page 7 |  |
| Credentials | 2 | What were the researcher’s credentials? E.g., PhD, MD | Additional file 2 |  |
| Occupation | 3 | What was their occupation at the time of the study? | Additional file 2 |  |
| Gender | 4 | Was the researcher male or female? | Page 8 & Additional file 2 |  |
| Experience and training | 5 | What experience or training did the researcher have? | Page 7 |  |
| Relationship with participants | | | |  |
| Relationship established | 6 | Was a relationship established prior to study commencement? | Page 6 |  |
| Participant knowledge of  the interviewer | 7 | What did the participants know about the researcher? e.g., personal  goals, reasons for doing the research | Page 6 |  |
| Interviewer characteristics | 8 | What characteristics were reported about the inter viewer/facilitator? e.g., Bias, assumptions, reasons and interests in the research topic | Page 8-9 & Additional file 2 |  |
| Domain 2: Study design | | | |  |
| Theoretical framework | | | |  |
| Methodological orientation  and Theory | 9 | What methodological orientation was stated to underpin the study? e.g. grounded theory, discourse analysis, ethnography, phenomenology, content analysis | Page 9 |  |
| Participant selection | | | |  |
| Sampling | 10 | How were participants selected? e.g., purposive, convenience, consecutive, snowball | Page 6 |  |
| Method of approach | 11 | How were participants approached? e.g., face-to-face, telephone, mail, email | Page 6 |  |
| Sample size | 12 | How many participants were in the study? | Pages 11 |  |
| Topic | Item No. | Guide Questions/Description | Reported on  Page No  . | |
| Non-participation | 13 | How many people refused to participate or dropped out? Reasons? | N/A |  |
| Setting | | | |  |
| Setting of data collection | 14 | Where was the data collected? e.g., home, clinic, workplace | Pages 8 & 25 |  |
| Presence of non-  participants | 15 | Was anyone else present besides the participants and researchers? | N/A |  |
| Description of sample | 16 | What are the important characteristics of the sample? e.g., demographic data, date | Page 10-11 |  |
| Data collection | | | | |
| Interview guide | 17 | Were questions, prompts, guides provided by the authors? Was it pilot tested? | Additional file 1 | |
| Repeat interviews | 18 | Were repeat inter views carried out? If yes, how many? | N/A | |
| Audio/visual recording | 19 | Did the research use audio or visual recording to collect the data? | Page 8 | |
| Field notes | 20 | Were field notes made during and/or after the interview or focus group? | Page 8 | |
| Duration | 21 | What was the duration of the interviews or focus group? | Page 10 | |
| Data saturation | 22 | Was data saturation discussed? | Page 7 | |
| Transcripts returned | 23 | Were transcripts returned to participants for comment and/or correction? | N/A | |
| Domain 3: Analysis and findings | | | | |
| Data analysis | | | | |
| Number of data coders | 24 | How many data coders coded the data? | Pages 8-9 | |
| Description of the coding  tree | 25 | Did authors provide a description of the coding tree? | Pages 8-9 | |
| Derivation of themes | 26 | Were themes identified in advance or derived from the data? | Pages 8-9 | |
| Software | 27 | What software, if applicable, was used to manage the data? | Page 8 | |
| Participant checking | 28 | Did participants provide feedback on the findings? | N/A | |
| Topic | Item No. | Guide Questions/Description | Reported on  Page No  . | |
| Reporting | | | | |
| Quotations presented | 29 | Were participant quotations presented to illustrate the themes/findings?  Was each quotation identified? e.g., participant number | Pages 12-23 | |
| Data and findings consistent | 30 | Was there consistency between the data presented and the findings? | Pages 12-23 | |
| Clarity of major themes | 31 | Were major themes clearly presented in the findings? | Figure 1 & Pages 12-23 | |
| Clarity of minor themes | 32 | Is there a description of diverse cases or discussion of minor themes? | Page 23-24 | |
