# Supplementary material for: “I want to see them thrive!”: exploring health service research priorities for young Aboriginal children growing up in Alice Springs – a qualitative study
Source: BMC Health Serv Res. 2024 Feb 15;24:205. doi: 10.1186/s12913-024-10642-8 (PMC10868103; doi:10.1186/s12913-024-10642-8)
Supplement: Supplementary file 4 — Additional file 4. [file 12913_2024_10642_MOESM4_ESM.docx]

# **ADDITIONAL FILE 4**

**“I want to see them thrive!”: exploring health service research priorities for young Aboriginal children growing up in Alice Springs – a qualitative study”**

**CONSOLIDATED CRITERIA FOR STRENGTHENING REPORTING OF HEALTH RESEARCH INVOLVING INDIGENOUS PEOPLES: THE CONSIDER STATEMENT**

Huria, T., Palmer, S.C., Pitama, S. *et al.* Consolidated criteria for strengthening reporting of health research involving indigenous peoples: the CONSIDER statement. *BMC Med Res Methodol* **19**, 173 (2019). <https://doi.org/10.1186/s12874-019-0815-8>

| **Item Checklist Item** | | **Reported on Page No.** |  |  |
| --- | --- | --- | --- | --- |
| Governance | |  |  |  |
| 1. | Describe partnership agreements between the research institution and Indigenous-governing organization for the research, (e.g., Informal agreements through to MOU (Memorandum of Understanding) or MOA (Memorandum of Agreement)). | A Collaborative Research Agreement was drafted between Congress and the Murdoch Children’s Research Institute. This agreement outlined the roles and responsibilities of both parties in working together to complete the project. It also included protection of Indigenous intellectual property and knowledge arising from the research, including financial and intellectual benefits generated. This agreement was executed by executive representatives from both institutes on the 23rd of April 2020.  This partnership is described on page 5 of the manuscript. |  |  |
| 2. | Describe accountability and review mechanisms within the partnership agreement that addresses harm minimization. | Accountability and review mechanisms have been built into the study protocol and the joint collaborative research agreement: “The Study will be guided by the responsibility to do no harm to the participants, participating organisations or the local or wider community”. A risk management plan is outlined in the Study protocol.  This is referred to on page 8: “participants social and emotional safety were closely monitored as per protocol”. |  |  |
| 3. | Specify how the research partnership agreement includes protection of Indigenous intellectual property and knowledge arising from the research, including financial and intellectual benefits generated (e.g., development of traditional medicines for commercial purposes or supporting the Indigenous community to develop commercialization proposals generated from the research). | The standard terms of the NHMRC that applies to ownership of intellectual property apply to this project. The intellectual property will be vested in the communities, investigators and the institutions to which they belong. The research agreement established between MCRI/ UoM & Congress will ensure they jointly own the intellectual property. The project is a public good initiative, and all efforts will be made to ensure the outcomes of research are directed to prioritising the public good. The partners have jointly negotiated a plan regarding the use and benefits of any materials or knowledge used or created through this research to support the protection of Aboriginal intellectual property. The research agreement contains provisions for any articles, materials or public presentations arising from the project be reviewed and approved by representatives from the Congress Research Sub-Committee prior to publication.  The project has no scope for commercial benefit. The approved ethics application included reference to how cultural knowledge would be managed and protected throughout the project. |  |  |
| Prioritization | |  |  | |
| 4. | Explain how the research aims emerged from priorities identified by either Indigenous stakeholders, governing bodies, funders, non-government organization(s), stakeholders, consumers, and empirical evidence | During a research planning meeting in 2013, Congress members called for more research to focus on the health and wellbeing of young Aboriginal children as they grow. This request for more longitudinal data was the genesis for the partnership proposal [between Congress, MCRI & UoM] to look at the feasibility of establishing a longitudinal cohort study of young Aboriginal children in Alice Springs.  In order to address the scarcity of literature that documents the real-world experiences of researchers working on longitudinal studies of Indigenous children, we embarked on the current descriptive qualitative study.  This is described on page 5 of the manuscript. |  |  |
| Relationships (Indigenous stakeholders/participants and Research team) | |  |  | |
| 5. | Specify measures that adhere and honor Indigenous ethical guidelines, processes, and approvals for all relevant Indigenous stakeholders, recognizing that multiple Indigenous partners may be involved, e.g., Indigenous ethics committee approval, regional/national ethics approval processes. | The current study was endorsed by the Congress Research Sub-Committee. Aboriginal leaders are represented on the Congress Research Sub-Committee which exists to support and promote research that is responsive to the needs of the local Aboriginal community in Alice Springs. The project also underwent separate ethical review by the Royal Children’s Hospital Human Ethics Research Committee (2019.155) and Central Australian Human Research Ethics Committee (CA-19-3519). The research was conducted in accordance with various ethical guidelines for conducting research involving Aboriginal and Torres Strait Islander communities. |  |  |
| 6. | Report how Indigenous stakeholders were involved in the research processes (i.e., research design, funding, implementation, analysis, dissemination/recruitment). | Project design was conducted in consultation with Congress Aboriginal staff and with input from a senior Aboriginal academic at the UoM. Funding was applied for by the same senior Aboriginal academic at UoM. A collaborative team of Aboriginal and non-Aboriginal researchers working together on the analysis, interpretation and final dissemination of results. |  |  |
| 7. | Describe the expertise of the research team in Indigenous health and research. | The first author (CLJ) is a non-Aboriginal doctoral researcher who grew up on Wathawurrung country in regional Victoria. CLJ is a mother of two children who has worked on early childhood research since 2005 but only in the past 5 years has worked in the field of Aboriginal health. Before commencing work in Alice Springs, the first author completed several short courses focusing on cross cultural communication, cultural safety, and Aboriginal culture in Central Australia. Cultural oversight and mentorship were provided to CLJ by authors SE & AH. CLJ engaged in continuous two way learning with her fellow co-authors and partners at Congress over the course of the project.  AH was born in Alice Springs and has lived most of her life throughout the NT. She is a Yangkunjatjarra woman with familial ties throughout the NT and northern SA.  She works at Congress as the NDIS Support Team Leader and is educated as a social worker. Angela previously worked in the Intensive Family Support Program at Congress providing practical and therapeutic support for vulnerable Aboriginal families for 8 years. Angela led fieldwork for the Healthy Journey for Kids research project in 2020 and continues on as a co-author and Associate Investigator.  SG & AD are both paediatricians. SG is a professor at the MCRI where her research focuses on investigating, testing, and translating sustainable policy relevant solutions that eliminate inequities for children. AD is an associate professor at the University of Melbourne and has lived and worked in the Northern Territory. Her doctoral work led to the creation of the of the ASQ-TRAK developmental screening tool – the first culturally appropriate tool for use with Australian Aboriginal and Torres Strait Islander children. SG, SE & AD are all based in Melbourne, Victoria, but their research network extends across much of Australia.  SE is a Noongar woman from Southwest WA and the first Aboriginal medical doctor to be awarded a PhD in 2003. SE is an experienced epidemiologist who was recently awarded an OA in 2022 for "distinguished service to medical research, to Indigenous health, and to professional organisations". She leads many epidemiological studies including projects based in Central Australia. |  |  |
| Methodologies | |  |  |  |
| 8. | Describe the methodological approach of the research including a rationale of methods used and implication for Indigenous stakeholders, e.g., privacy and confidentiality (individual and collective) | The current study is informed by phenomenology seeking to explore participants’ lived experiences and rich insights to generate a better understanding issues affecting feasibility.  Moreover, our research is grounded in a transformative paradigm, which argues that knowledge reflects power relations in society, and seeks to rectify this imbalance by prioritizing local voices and needs.  The findings have been reported using a strengths-based approach that is inclusive of Indigenous values.  See page 10. |  |  |
| 9. | Describe how the research methodology incorporated consideration of the physical, social, economic and cultural environment of the participants and prospective participants. (e.g., impacts of colonization, racism, and social justice). As well as Indigenous worldviews. | There was direct engagement between partners and local Aboriginal researchers in the study design, data collection and analysis phases. The collaborative relationships built between partners ensured that engagement was ongoing and allowed for flexibility in adapting research activities to meet local needs and protocols. This resulted in minor modifications to the research plan submitted to the relevant HRECs for approval. Capturing the voices of local Aboriginal caregivers was critical for us, as such we used purposive sampling approach to recruit as many Aboriginal participants as possible. |  |  |
| Participation | |  |  |  |
| 10. | Specify how individual and collective consent was sought to conduct future analysis on collected samples and data (e.g., additional secondary analyses; third-parties accessing samples (genetic, tissue, blood) for further analyses). | N/A |  |  |
| 11. | Described how the resource demands (current and future) placed on Indigenous participants and communities involved in the research were identified and agreed upon including any resourcing for participation, knowledge, and expertise | The project was initiated with philanthropic funds secured from John T Reid Charitable Trustees. These funds provided employment for two local Aboriginal researchers over a period of time and resourcing community participants. Author AH was provided with funds/capacity to travel interstate to attend a conference to co-present research findings. |  |  |
| 12. | Specify how biological tissue and other samples including data were stored, explaining the processes of removal from traditional lands, if done, and of disposal. | A data management plan was devised as part of the Collaborative Research Agreement between Congress & MCRI. Data was held at Congress on password protected files on a locally hosted server that was shared securely with MCRI researchers. |  |  |
| Capacity | |  |  |  |
| 13. | Explain how the research supported the development and maintenance of Indigenous research capacity (e.g., specific funding of Indigenous researchers). | As stated above Item 11. |  |  |
| 14. | Discuss how the research team undertook professional development opportunities to develop the capacity to partner with Indigenous stakeholders? | As stated above Item 7 and in Additional file 2. |  |  |
| Analysis and interpretation | |  |  |  |
| 15. | Specify how the research analysis and reporting supported critical inquiry and a strength-based approach that was inclusive of Indigenous values. | Data analysis for the current study involved the contributions and interpretations of Aboriginal academics (ES, AH & SE). Findings were reported using a strengths-based approach inclusive of Indigenous values. This was an essential step to ensure that the knowledge synthesised and presented was interpreted in a culturally appropriate manner. |  |  |
| Dissemination | |  | |  |
| 16. | Describe the dissemination of the research findings to relevant Indigenous governing bodies and peoples. | Dissemination of findings from this feasibility study will be subject to approval by the Congress Research Sub-Committee in accordance with the Collaborative Research Agreement. With approval, findings will be disseminated through: community meetings/workshops, community reports, fact sheets, manuscripts for peer-reviewed publication, conference presentations, public seminars, flipbooks, data visualisation and video and public seminars. In addition, there is a clear obligation under research guidelines for working with Aboriginal and Torres Strait Islander communities to report results back to participants involved. This will occur through the production and dissemination of plain language reports and diagrams and presentations to communities. Budget will be sought for production of an explainer video. |  |  |
| 17. | Discuss the process for knowledge translation and implementation to support Indigenous advancement (e.g., research capacity, policy, investment). | The direct and active involvement of key stakeholders including Congress, Aboriginal community members, practitioners, service providers, and policy organisations, will ensure that the study’s research questions and outcomes address issues that are both relevant to, and a priority for, local Aboriginal people. It is our hope that the synthesis, dissemination of knowledge generated from this study (and the future cohort study) will influence, change and even improve the way in which data is used by Congress to track the health and development of local Aboriginal children moving forward. |  |  |
